# Exploring potent aldose reductase inhibitors for anti-diabetic (anti-hyperglycemic) therapy: integrating structure-based drug design, and MMGBSA approaches

**DOI:** 10.3389/fmolb.2023.1271569

**Published:** 2023-11-20

**Authors:** Muhammad Shahab, Guojun Zheng, Fahad M. Alshabrmi, Mohammed Bourhia, Gezahign Fentahun Wondmie, Ahmad Mohammad Salamatullah

**Affiliations:** ^1^ State Key Laboratories of Chemical Resources Engineering Beijing University of Chemical Technology, Beijing, China; ^2^ Department of Medical Laboratories, College of Applied Medical Sciences, Qassim University, Buraydah, Saudi Arabia; ^3^ Department of Chemistry and Biochemistry, Faculty of Medicine and Pharmacy, Ibn Zohr University, Agadir, Morocco; ^4^ Department of Biology, College of Science, Bahir Dar University, Bahir Dar, Ethiopia; ^5^ Department of Food Science and Nutrition, College of Food and Agricultural Sciences, King Saud University, Riyadh, Saudi Arabia

**Keywords:** aldose reductase, diabetes melitus, SBVS, molecular docking simulation, MMGBSA

## Abstract

Aldose reductase (AR) is an important target in the development of therapeutics against hyper-glycemia-induced health complications such as retinopathy, etc. In this study, we employed a combination of structure-based drug design, molecular simulation, and free energy calculation approaches to identify potential hit molecules against anti-diabetic (anti-hyperglycemic)-induced health complications. The 3D structure of aldoreductase was screened for multiple compound libraries (1,00,000 compounds) and identified as ZINC35671852, ZINC78774792 from the ZINC database, Diamino-di nitro-methyl dioctyl phthalate, and Penta-o-galloyl-glucose from the South African natural compounds database, and Bisindolylmethane thiosemi-carbazides and Bisindolylme-thane-hydrazone from the Inhouse database for this study. The mode of binding interactions of the selected compounds later predicted their aldose reductase inhibitory potential. These com-pounds interact with the key active site residues through hydrogen bonds, salt bridges, and π-π interactions. The structural dynamics and binding free energy results further revealed that these compounds possess stable dynamics with excellent binding free energy scores. The structures of the lead inhibitors can serve as templates for developing novel inhibitors, and *in vitro* testing to confirm their anti-diabetic potential is warranted. The current study is the first to design small molecule inhibitors for the aldoreductase protein that can be used in the development of therapeutic agents to treat diabetes.

## Introduction

Aldo-Keto-Reductases (AKRs) are versatile enzymes involved in metabolizing carbonyl-containing substrates like sugars, lipid aldehydes, ketosteroids, and keto prostaglandins (Bohren et al., 1989; Komoto et al., 2004). This superfamily comprises 16 families, ranging from AKR1 to AKR16 (Komoto et al., 2004), that share a high degree of sequence similarity and a common protein folding structure. The AKR website (http://www.med.upenn.edu/akr/) provides information about the AKR superfamily. AKR enzymes exhibit similar catalytic and structural properties and are NAD(P)H-dependent oxidoreductases expressed as 34–37 kDa polypeptides (Jez et al., 1997). The AKR1 is further categorised into the A to E subfamilies; among them, AKRB1 is comprehensively studied and plays a major role in the emergence of diabetic complications (Blakeley et al., 2008). These enzymes make up the “polyol pathway,” an alternative glucose metabolism process that runs concurrently with glycolysis and causes hyperglycemia in diabetic patients (Singh et al., 2021). Hyperglycemia-induced pathways drive oxidative stress in diabetic organs (heart, kidney, and eye) through AGEs, the polyol pathway, the mitochondrial electron transport system, and PKC activation (Srivastava et al., 2005; Bhatnagar and Srivastava, 1992). In metabolic processes like the glutathione reductase/glutathione peroxidase system’s detoxification of reactive oxygen species (ROS), NADPH plays reductive roles (Paul et al., 2020). An increased cytosolic NADH/NAD + ratio causes mitochondrial NADH-dependent pathways, which induce ROS (Vedantham et al., 2012) Increased NADH may also ameliorate the production of diacylglycerol (DAG), which activates PKC and causes oxidative stress by activating NAD(P)H oxidase by regulating PKC. The prevalence of diabetes has been rising alarmingly worldwide. According to the World Health Organization (https://www.who.int/health-topics/diabetes), more than 400 million people worldwide are currently suffering from diabetes. As a result, diabetes complications have risen in tandem with the rise in the number of people with the disease (Taslimi et al., 2018; Demir et al., 2020). Cardiovascular disease (CVD) is the main cause of morbidity and mortality in people with diabetes mellitus, among the different diabetic complications (Jandeleit-Dahm and Cooper, 2002). Diabetic cardiomyopathy is a unique cardiovascular condition characterized by impaired cardiac function in individuals with diabetes that is unrelated to coronary artery disease (Sower et al., 2001; Lopaschuk, 2002), Diabetes increases myocardial sensitivity to ischemia and raises the risk of cardiovascular disease and myocardial infarction (Lehto et al., 1994; Stone et al., 1995). Cardiovascular dysfunction in diabetics has been attributed to increased sorbitol buildup and a decline in NADPH due to an AR flux (Flores et al., 2023). Diabetic patients demonstrate a heightened incidence of cardiovascular disease and myocardial infarction (Jhuo et al., 2022). Cardiac dysfunction in diabetic patients is attributed to increased sorbitol accumulation and reduced NADPH levels resulting from aldose reductase (AR) flux (De Geest and Mishra, 2022; Garg and Gupta, 2022). According to studies, AR activation causes oxidative stress, (Ramana et al., 2006b; Ramana and Srivastava, 2006), which in turn can activate the NF-κB pathway (Ramana et al., 2006a; Ramana et al., 2006b; Ramana and Srivastava, 2006). Additionally, by lowering oxidative stress, AR inhibition can prevent acute hyperglycemia-induced cardiac contractile dysfunction (Aly et al., 2023). The blocking of the NF-κB pathway and oxidative stress by AR suppression is a new strategy for avoiding cardiovascular diseases.

## Materials and methods

### Preparations of protein structure

The 3D structure of an enzyme aldose reductase with (PDB ID; 3S3G) was extracted by utilizing the PDB database (Zheng et al., 2012). The three-dimensional structure was further checked for chain breaks, and missing atoms and water molecules were extracted. Furthermore, the retrieved protein structure was subjected to preparatory procedures using the Dock prep module of UCSF Chimera v1.10.2 software program (Goddard et al., 2007). The partial charges were used, which properly set the protein model’s protonation phase at a neutral pH. The GBVI/WSA rescoring approach and London dG scoring function were also utilised in combination with the Triangle matcher docking algorithm. Finally, a protein-ligand interaction fingerprint was used to figure out hydrophobic bonds, ionic bonds, and hydrogen bonds (Méndez-Álvarez et al., 2023). In addition, the three-dimensional structure of the standard drug (tolmetin) was retrieved from the Pub Chem database. The structure was energy-minimised and prepared by AutoDock Vina.

### Preparation of commercial and in house databases

Virtual screening was conducted using three databases: the ZINC database (Ghufran et al., 2022) (1 million compounds), the South African Natural Compounds database (available at http://african-compounds.org/about/afrodb/), and with the help of the research of our collaborators, the three-dimensional structures of the compounds were put into a database called an In-house containing 1,600 compounds. These databases were utilized to identify highly active and potent inhibitors against aldose reductase. LogP, LogS, Lipinski’s, Pfizer, GSK, and the Golden Triangle rules were among the properties predicted for the finally selected hits. Subsequently, aldose reductase screening was performed on the compounds passing the RO5 criteria from each database, utilizing scoring and Minimization with AutoDock Vina (smina) (Ding et al., 2023). Following Smina screening, the top hits were subjected to further evaluation using ADFR. ADFR employed flexible docking with high accuracy for each compound against aldose reductase, utilizing the AutoDock four scoring function (Aly et al., 2023). Finally, from in-house and commercial databases (ZINC and the South African Natural Compounds Database), the top two hits were finalized from each database on the basis of docking score and binding interaction using Schrodinger Maestro and Pymol software and carried out for MD simulation (Maya Díaz, 2023; Taherkhani et al., 2023).

### Screening of libraries

The structure base Virtual screening uses the three-dimensional structure of ligands and proteins in the database. In order to determine potent ligands, we carried out molecular docking to assess the methods by which ligands and proteins bind. This approach predicts the beneficial and enhanced interactions between proteins and ligands. In addition, SANCDB, an in-house database, and ZINC databases The drug libraries, including the ZINC database, the in-house database, and SANCDB, were screened by the SBVS using AutoDock 4. AutoDock 4, a docking suite, was utilised to screen the compounds obtained from SANCDB, ZINC, and the in-house databases, employing a homology model. To ensure the robustness and reliability of our molecular docking analysis, we employed three distinct servers: Glide module software (Schrödinger Maestro v12.1) (Schrödinger, 2011; Kaushik et al., 2018). These servers are renowned for their user-friendly interfaces and accuracy in delivering reliable docking results. This multipronged approach enhances the reliability of our findings and strengthens the scientific rigor of our study also it allowed us to assess the consistency and reliability of our docking scores. The model’s protonation phase was appropriately adjusted for neutral pH, and partial charges were included. The London dG scoring function, Triangle Matcher Docking algorithm, and GBVI/WSA rescoring method were employed. Additionally, a force field-based scoring function was utilized for post-docking refinement. Thereafter, the interaction of each ligand with the active site was determined by utilizing the protein-ligand interaction fingerprint in AutoDock 4. PLIF determines how hydrogen atoms, water molecules, and ions interact with each other (Adessi et al., 2023). Finally, MD simulation was performed in order to verify the molecular docking approaches.

### Molecular dynamic simulation

A molecular dynamic (MD) simulation was carried out in order to evaluate the dynamic behavior of receptors with inhibitors at the atomic level. On the basis of binding interactions and docking scores, the best hits were used to perform MD simulation, and free energy calculation-based validation was carried out by using AMBER22 (Case et al., 2005; Shahab et al., 2023a). Initially, the drug topology was created by utilizing the parmchk2 and antechamber (Khan et al., 2023). Thereafter, all complexes obtained were constructed using the Tleap preparation programme. An octahedral box was used, and by introducing the Na + or Cl-ions, all complexes were neutralised. To prepare the complexes, topology and coordinate files were used for a two-stage minimization process: 1) 12,000 steps for the first round and 2) 6,000 steps for the second round. Subsequently, each complex underwent heating and equilibration for 20 ns In the production stage, a 100 ns simulation was conducted. For accelerated MD simulation, the GPU version of PMEMD.cuda was employed. Trajectories obtained were processed using the CPPTRAJ and PTRAJ tools (Jama et al., 2023; Wang et al., 2023).

### Binding free energy evaluation

The use of the MMPBSA.py script in the MD simulation run, trajectories were identified, which were further used in calculating the binding free energy (Shahab et al., 2023b). For calculating the binding free energy of any complexes, including protein-ligand, nucleic acid-protein, and protein-protein, this type of approach was used (Khan et al., 2023). Hence, we also applied this approach here to accurately compute the total binding free energy of the protein-ligand complexes. Mathematically the binding free energy can be estimated as:

″∆G bind=G complex−Greceptor+Gligand″

Different contributing components of total binding energy were calculated by the following equation:

″G=Gbond+Gele+GvdW+Gpol+Gnpol″

G_bond_, G_electrostatic_, and G_vdW_ refer to interactions involving bonded, electrostatic, and van der Waals states, respectively. On the other hand, G_polar_ and G_npolar_ describe polar and non-polar interactions, respectively, which are calculated based on the assumed free energy through precise Generalized Born (GB) methods (Chen et al., 2019; Chen et al., 2022).

### Prediction of bio activity and dissociation constant (K_D_)

The bio-activity and dissociation constant (KD) were computationally predicted for top hits by utilizing an online web server, Molinspiration (https://www.molinspiration.com/cgi-bin/properties) and PRODIGY-Ligand (https://wenmr.science.uu.nl/prodigy/lig) respectively (Vangone et al., 2019). These online tools were reported previously to demonstrate the bioactivity and KD of various molecules against diseases (Khan et al., 2021).

## Results and discussion

Aldose reductase, also known as AKR1B1, is an enzyme belonging to the aldo-keto reductase family. It relies on NADPH as a cofactor and is responsible for catalyzing the reduction of both hydrophilic and hydrophobic aldehydes. It serves as the initial enzyme in the polyol pathway, which converts glucose into sorbitol. Sorbitol is then further metabolized to fructose by the action of sorbitol dehydrogenase. The activation of the polyol pathway, particularly in hyperglycemic conditions, is widely accepted as the key event leading to various long-term complications associated with diabetes. Due to the significant role of AKR1B1 in the development of diabetic complications, researchers have targeted this enzyme for the development of molecules that can inhibit its activity (Balestri et al., 2022). In our study, the computational analysis revealed that the identified compounds form specific interactions with key amino acids within the active site of aldose reductase, such as Ser302, Phe122, Trp219, Cys298, Ala299, Val297, and Trp20 and Leu300 integrating structure-based drug design (SBDD) and molecular mechanics/generalized born surface area (MMGBSA) approaches (Kinoshita, 1990; Ramachandran et al., 2023). For instance, these residues are also reported to act as inhibitor hotspots for other drug targets (Fang et al., 2023).The topmost active ligands from each database (South African, ZINC, and in-house) based on docking results and interaction analysis were further subjected to validation through molecular dynamics (MD) simulations. Post-MD analyses were performed to assess the ligands’ behaviour and proper-ties. Therefore, the present study used structure-based virtual screening, molecular dynamic simulation, and binding free energy approaches to determine potent inhibitors against aldose reductase. The combination of these computational techniques facilitated the rational design and prioritization of compounds based on their predicted binding energies and structural characteristics. In various computational approaches, including structure-based virtual screening and MD simulation, great concern is given to accelerating the cycle of drug development. The identified inhibitors hold promise for further development and experimental evaluation, potentially leading to novel treatments for hyper-glycemic patients and addressing the complications associated with diabetes. I believe this research offers promising insights into potential aldose reductase inhibitors for managing hyperglycemia and diabetes-related complications, it is important to acknowledge its limitations. The findings are primarily based on computational methods and simulations, specifically structure-based drug design (SBDD) and molecular mechanics/generalized born surface area (MMGBSA) approaches. These predictions, although promising, necessitate rigorous experimental validation to assess the efficacy, safety, and specificity of the identified compounds in biological systems. Additionally, the study does not address critical aspects such as pharmacokinetics, potential off-target effects, and the variability among diverse patient populations. Recognizing these limitations is crucial to offer a balanced and realistic perspective on the study’s implications and the potential for clinical translation of the findings.

### Molecular docking

#### Analyzing binding modes of top hits retrieved from african medicines database

**FIGURE 1:**
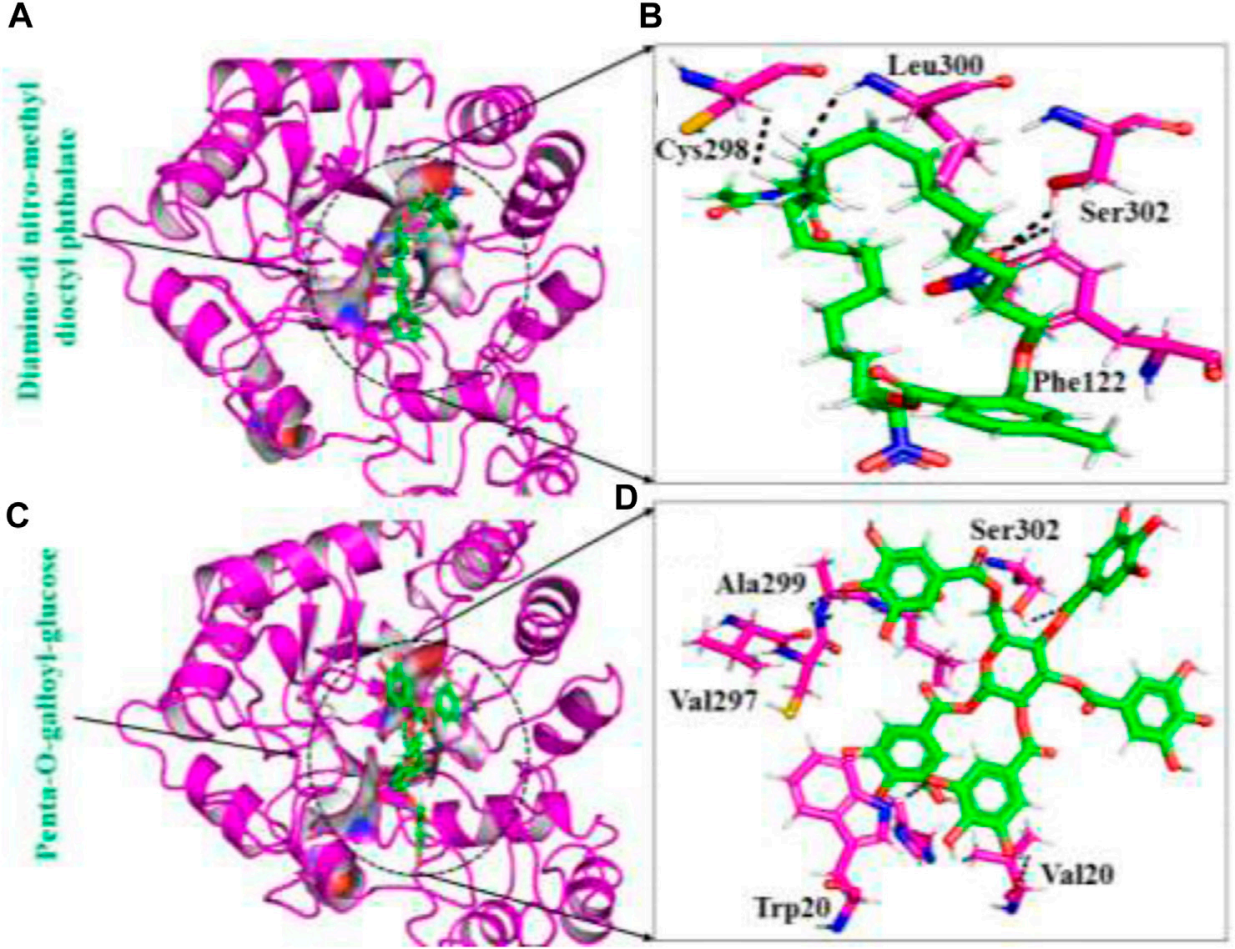
**(A)** The binding pocket of diamine-dinitro-methyl dioctyl phthalate with the aldoreductase receptor **(B)** the binding interaction pattern of the diamine-dinitro-methyl dioctyl phthalate demonstrating the key interacting residues. **(C)** The binding pocket of penta-O-galloyl-glucose with the aldoreductase receptor **(D)** the binding interaction pattern of the penta-O-galloyl-glucose, demonstrating the key interacting residues.

The South African Natural Compounds Database (SANCDB) is a valuable resource for various disease treatments. Screening SANCDB identified diamine-dinitro-methyl dioctyl phthalate and penta-o-galloyl-glucose as the top hits among the 570 compounds. These compounds demonstrated docking scores of -12.24 kcal/mol and -11.34 kcal/mol, respectively. Then we validated molecular docking score by Schrödinger Maestro v12.1 (−12.265, −11.532 kcal/mol). In terms of interaction, Diamino-di nitro-methyl dioctyl phthalate established five hydrogen bonds with Ser302, Phe122, Trp219, Cys298, and Leu300 residues Figures 1A, B. The interacting residues observed in the South African Natural Compounds Database (SANCDB) align with the compounds reported in the ZINC database. The binding residues, including Trp20, Cys298, Tyr309, and Asn260, were found to be involved in the interaction with the compounds. Additionally, a π-π interaction was established with the Tyr209 residue. These findings indicate consistent and specific interactions between these small molecules and the critical residues of aldoreductase. The compound penta-o-galloyl-glucose established four hydrogen bonds involving Ala299, Val297, Ser302, and Trp20 (Figures 1C, D). Diamino-di nitro-methyl dioctyl phthalate was found to interact with aldose reductase through hydrogen bonds, exhibiting a similar interaction pattern to the compounds reported in the ZINC and Inhouse databases in this study. This suggests potential pharmacological activity against aldose reductase. The interaction patterns of each compound and their respective docking scores are presented in Table 1.

**TABLE 1:** Protein-ligand interaction details and their docking of the standard drug and final hits score.

Compound	Docking score		Interaction details							
			Ligand		Receptor			Interaction	Distance	E(kcal/mol)
*African database (A)*										
*Diamino-di nitro-methyl dioctyl phthalate*		−12.24	C	1	SG	CYS	298	H-donor	3.80	−0.2
			O	3	CD1	LEU	300	H-acceptor	3.87	−1.5
			O	17	CD1	LEU	300	H-acceptor	3.52	−0.2
			O	22	OG	SER	302	H-acceptor	3.09	−6.4
			O	17	CD1	PHE	122	H-acceptor pi-H	3.76	−3.3
			C	7	6-ring	TRP	219	pi-H	3.65	−1.3
			C	5	6-ring	TRP	20		4.33	−2.1
*Penta-O-galloyl-glucose*		−11.34	O	6	SG	CYS	122	H-donor	3.16	−1.4
			O	28	O	VAL	297	H-donor	2.86	−1.6
			C	31	6-ring	PHE	122	H-pi pi-H	4.13	−0.7
			6-ring		CB	PHE	122		3.67	−0.8
*In-house database (B)*										
*Bisindolylmethane thiosemi-carbazides*		−10.25	N	7	O	VAL	47	H-donor	3.06	−0.5
			S	16	O	ASP	216	H-donor	4.06	−0.1
			C	19	O	ASP	216	H-donor	3.55	−0.2
			C	19	OD2	ASP	216	H-donor	3.77	−0.2
			C	45	O	CYS	298	H-donor	4.27	−0.1
			S	16	CD	PRO LEU	218	H-acceptor	4.48	−0.1
			O	19	CD1		300	H-acceptor	3.61	−0.1
Bisindolylmethane–hydrazone hybrids		−9.51	S	16	SG	CYS	298	H-donor	3.77	−0.7
			N	39	O	VL	47	H-donor	3.39	−0.8
			O	13	N	LEU	300	H-acceptor	3.09	−1.6
			S	16	NE1	TRP	111	H-acceptor	4.22	−0.0
			6-ring		6-ring	TRP	219	Pi-Pi	3.78	−0.7
*Zinc database (C)*										
*ZINC35671852*		−7.90	N5	22	CE	LYS	77	H-acceptor	3.38	−0.7
			C5		6-ring	TRP	20	H-pi	3.50	−0.5
			5-ring	8	6-ring	TYR	209	Pi-pi	3.19	−0.0
			6-ring		6-ring	TRP	219	Pi-pi	3.60	−0.0
ZINC78774792		−7.49	N3	18	CE	LYS	77	H-acceptor	3.45	−0.2
			O3		ND2	ASN	160	H-acceptor	3.03	−0.2
			6-ring		CA	LEU	300	H-pi	4.37	−0.2
			6-ring	19	CD1	LEU	300	Pi-pi	3.48	−0.1
			6-ring		6-ring	TYR	209	Pi-pi	3.32	−0.0
					6-ring	TRP	219		3.82	−0.0
*Control drug* (*Tolmetin*)		−6.71	O2	18	NE1	TRP	11	H-acceptor	3.16	−2.4
			O2	18	ND2	ASN	160	H-acceptor	2.69	−6.5
			O3	19	OH	TYR	48	H-acceptor	2.75	−0.5

#### Binding modes of top hits from the inhouse database

**FIGURE 2:**
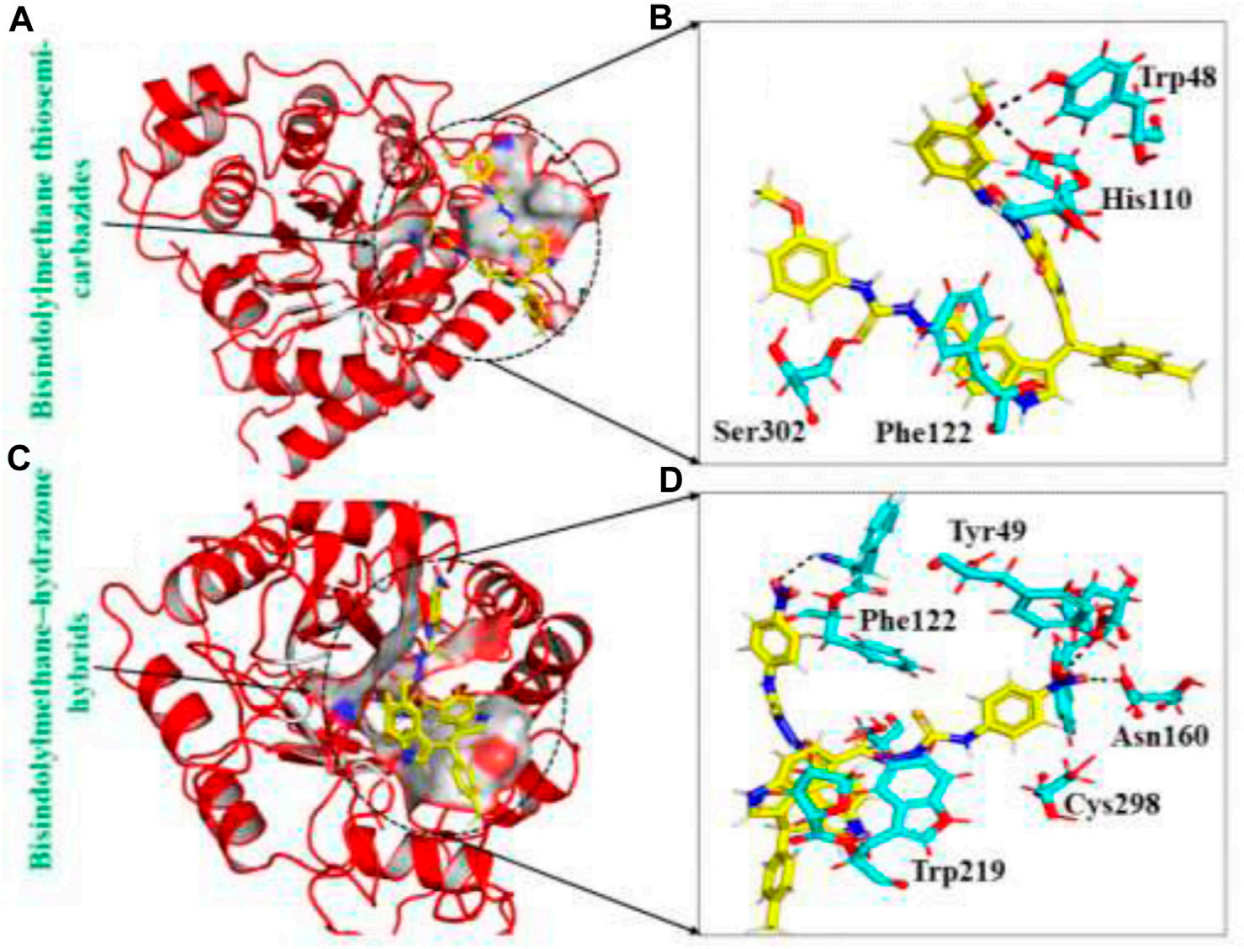
**(A)** The binding pocket of Bisindolylmethane thiosemi-carbazides with the aldoreductase receptor **(B)** the binding interaction pattern of the Bisindolylmethane thiosemi-carbazides, demonstrating the key interacting residues. **(C)** The binding pocket of Bisindolylmethane–hydrazone hybrids with the aldoreductase receptor **(D)** the binding interaction pattern of the Bisindolylmethane–hydrazone hybrids, demonstrating the key interacting residues.

The in-house database, containing over 1,300 derivatives of bis-indolylmethane, proves to be a valuable resource for designing natural product-based remedies for various diseases. Based on docking conformations, the ligands Bisindolylmethane thiosemi-carbazides and Bisindolylmethane-hydrazone hybrids were selected as top hits, demonstrating binding scores of -10.25 kcal/mol and -9.51 kcal/mol, respectively. Bisindolylmethane thiosemi-carbazides formed two hydrogen bonds with Trp48 and His110, while Bisindolylmethane-hydrazone hybrids formed three hydrogen bonds with Phe122, Tyr49, and Asn160. The docking score for Bisindolylmethane against the aldoreductase protein was reported as -10.25 kcal/mol, with key interactions involving Phe122, Tyr49, and Asn160. To validate the molecular docking score, we used Schrödinger Maestro v12.1, which yielded scores of -10.422 and -10.827 kcal/mol. The compounds identified in this particular database have smaller molecular structures and show promising pharmacological activity against aldoreductase. The interaction patterns of each compound are depicted in Figures 2A–D, and their respective docking scores are presented in Table 1.

#### Binding modes of top hits from the ZINC database

**FIGURE 3:**
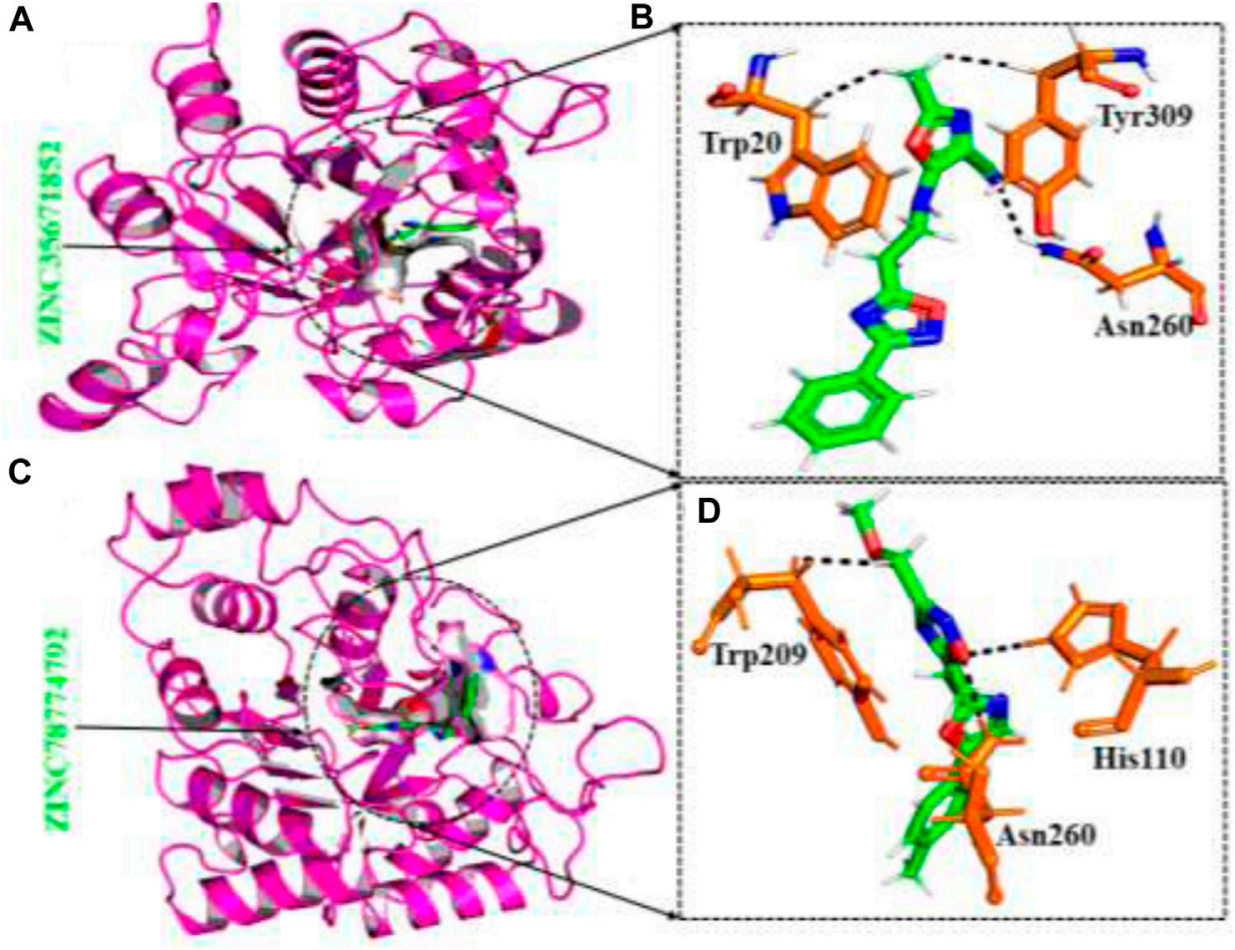
**(A)** The binding pocket of ZINC35671852 with the aldoreductase receptor **(B)** the binding interaction pattern of ZINC35671852, demonstrating the key interacting residues. **(C)** The binding pocket of ZINC78774792 with the aldoreductase receptor **(D)** the binding interaction pattern of the ZINC78774792 demonstrating the key interacting residues.

The ZINC database, comprising 100,000 druggable compounds, was screened, and 6,575 compounds were found to comply with the LogP, LogS, Lipinski’s, Pfizer, GSK, and Golden Triangle rules, which were among the properties predicted for the top two hits. Among these, 1,145 compounds were identified as the best hits. From these, two compounds, namely ZINC35671852 and ZINC78774792, were selected as the top hits based on their docking scores of -7.49 kcal/mol and -6.71 kcal/mol, respectively. To validate the molecular docking score, we used Schrödinger Maestro v12.1, which yielded scores of −8.753and -7.827 kcal/mol. ZINC35671852 formed three hydrogen bonds with the residues Trp20, Tyr309, and Asn260, while ZINC78774792 exhibited a similar hydrogen bond interaction pattern. These shortlisted compounds from the ZINC database displayed excellent docking scores and exhibited an interaction pattern that covered the entire active site, effectively blocking key residues and highlighting the pharmacological potential of these small molecules. The interaction patterns of each compound are depicted in Figures 3A–D, and their respective docking scores are presented in Table 1.

#### Molecular dynamic simulation

To properly understand the structure’s dynamic features, the binding of proteins with ligands is an essential parameter to reveal. Therefore, in the molecular dynamic simulation technique, we performed RMSD (structure stability), RMSF (residual flexibility), RoG, and hydrogen bond analysis to understand each ligand’s stability. The structure stability, which is calculated through RMSD as a function of time, shows that all ligands stably bind to the target protein except for some minor deviations.

#### Root mean square deviation

**FIGURE 4:**
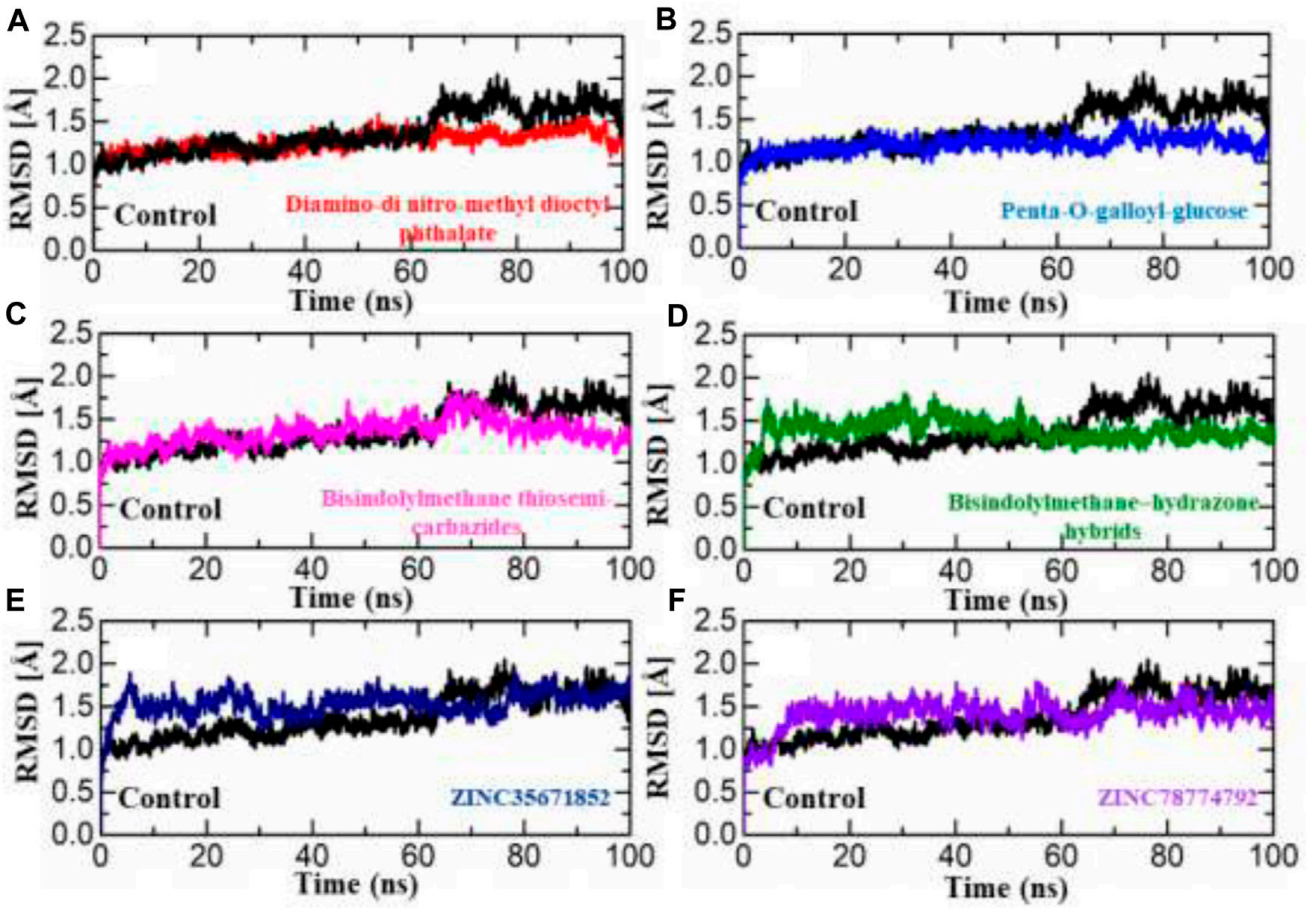
RMSD analysis was conducted to assess the dynamic stability of the hits from four databases in complex with aldoreductase. Figures **(A–F)** show the RMSD profiles for the hits from the African, Inhouse, and ZINC databases, respectively, compared to the control (Tolmetin-Aldoreductase). The results indicate that the hits exhibit lower RMSD values, suggesting more stable dynamics and promising interactions with aldoreductase.

The dynamic stability of a protein-ligand complex plays a crucial role in determining the pharmacological efficacy of a compound. A stable binding between the ligand and the protein’s active site indicates a higher potential for pharmacological activity. To evaluate the stability of the simulation trajectory, the Root Mean Square Deviation (RMSD) function is utilized in combination with simulation tools. In this study, the RMSD values for the trajectories of each complex were computed over time to assess their stability. The RMSD values for each complex are illustrated in Figures 4A–F. For this purpose, we carried out RMSd analysis to calculate the top two ligands from each database and the standard drug in the active site of aldose reductase. The RMSd analysis demonstrates that the top ligands exhibit stable behaviour but have minor deviations. The RMSd graph of the standard drug exhibits a small deviation within 0.5–1 Å up to 60 ns; thereafter, it increases from 1.2 to 1.7 Å up to 100 ns., ns and shows unstable behaviour (Figure 4). In the South African database, the SA1 complex exhibits significant stability throughout MD simulation from 0.6 to 0.7 Å till end of simulation (Figure 4A). Interestingly, the SA2 complex, according to the RMSd analysis presented, exhibits highly stable behaviour throughout the MD simulation period (average RMSD analysis 0.9Å (Figure 4B). In addition, for the compound Bisindolylmethane thiosemi-carbazides from the in-house database, RMSd graphs reveal stable behaviour at 1.0 to 1.3 Å at 65 ns; after that, the RMSd curve increased to 1.7 Å till 85 ns, thereafter reaching a stable state till the end of simulation (Figure 4C). Furthermore, Bisindolylmethane–hydrazonehybrids complex, the initial RMSd curve increases at 1.8 until 55 ns, then gradually decreases to 1.2, as presented in Figure 4D. In the Zinc database, initially the ZINC35671852 complex mediates an increase in the RMSd graph, reaching 1.9 Å up to 35 ns; thereafter, it gradually decreases to 1.5 Å and shows minor fluctuation till 100 ns (Figure 4E). Furthermore, the ZINC78774792 complex initially presents a decrease in the RMSd curve at 1.4 Å after reaching 55 ns, and the system shows minor fluctuations within 1.5–1.8 Å till end of simulation (Figure 4F). The control complex exhibited stable RMSD values, but the complexes formed with the novel compounds showed lower RMSD values, indicating more stable dynamics. This suggests that the novel compounds have a stronger and more stable interaction with aldoreductase. These findings highlight the potential therapeutic value of these compounds for the treatment of anti-diabetic (anti-hyperglycemic) conditions based on their interaction with aldoreductase.

#### Root mean square fluctuation (RMSF)

**FIGURE 5:**
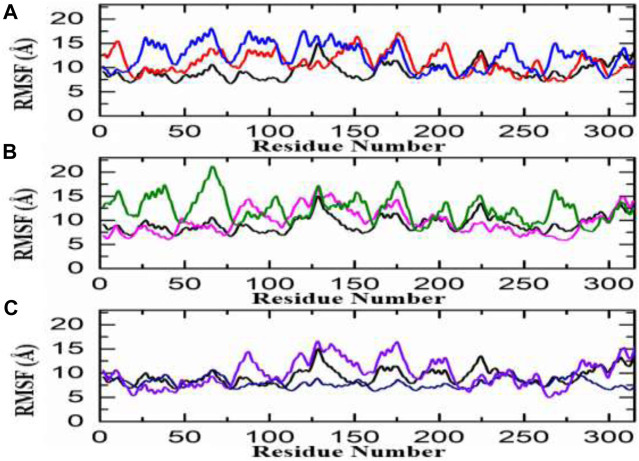
RMSf analysis of the finally selected hits **(A)** RMSf analysis Diamino-di nitro-methyl dioctyl phthalate/aldoreductase complex (red), Penta-O-galloyl glucose/aldoreductase complex (Blue) **(B)** Bisindolylmethane thiosemi-carbazides/aldoreductase complex (pink), Bisindolylmethane-hydrazone hybrids/Aldoreductase Complex (green) **(C)** ZINC35671852/Aldoreductase Complex (Purple), ZINC78774792/Aldoreductase Complex (Blue).

The calculation of residue flexibility has provided crucial information regarding molecular interaction patterns, inter-residue communication, protein coupling, inhibition potential, Biocatalysis, and enzyme engineering. The flexibility of each residue was evaluated and visually represented in Figures 5A–C. The fluctuations for each amino acid of aldose reductase in complex with ligands were evaluated through the RMSF curve, which accesses the stability of the active site towards compounds during the 100-ns MD simulation period. A lower number or reduced fluctuation signifies well-structured and less distorted regions within the complex. Interestingly, the flexibility of residues in the control complex exhibited similarities to the complexes of the top hits. Similarly, in the African database, all the compounds displayed a nearly identical pattern of residue flexibility. The region between 30 and 280 presented higher flexibility in the penta-o-galloyl-glucose complex only, while the regions between 10 and 120, 130–160, and 200–230 demonstrated higher fluctuation in Diamino-di nitro-methyl dioctyl phthalate complex in all the complexes (Figure 5A). Additionally, the compound Bisindolylmethane thiosemi carbazides exhibited significantly higher flexibility in the regions 75–180, while Bisindolylmethane-hydrazone hybrids showed higher flexibility in the regions 0–75, 80–110, and 230–280, as shown in Figure 5B. Moreover, a notable fluctuation was observed in the zinc database, where almost all regions displayed higher fluctuations except for 110–300. The RMSF graphs depicting the fluctuations in the zinc database complex are displayed in Figure 5B. Overall, these findings indicate that the binding of each ligand affects the internal dynamics in a different manner. It is noteworthy that the regions 160–190 encompass the active site residues, suggesting that the movement of this loop assists the drug in optimizing its position within the cavity by increasing the volume of the pocket. This ultimately facilitates the stable binding of ligands to the active site of aldose reductase.

#### Radius of gyration

**FIGURE 6:**
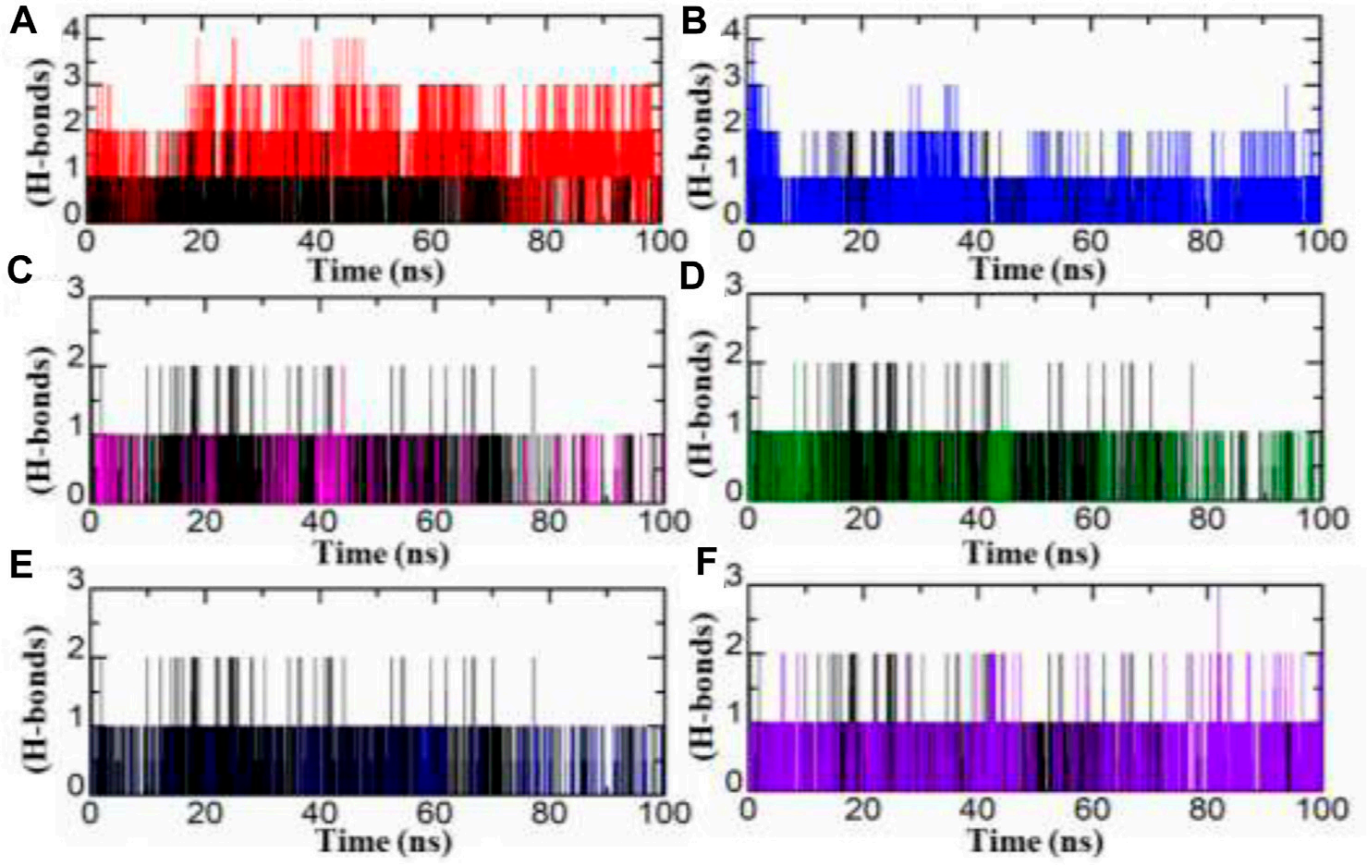
**(A–F)** Illustrating the analysis of hydrogen bond of standard drug (black) and the final hits.

The compactness of the system was evaluated by plotting the correlation between RoG (radius of gyration) and time. In comparison to conformational entropy, lower RoG values indicate a highly stable and compact structure, while higher RoG values indicate a lower degree of compactness in the structure. RoG is used to explore the folding and compactness of proteins; lower RoG values present strong compactness and strong structural rigidity, whereas high RoG values present less compactness and an unfolded state. The study of MD simulation presents the effects of inhibitors upon binding with proteins. As presented in Figure 6, RoG analysis shows that these obtained compounds from databases bound to aldose reductase, having lower RoG values as compared with standard drugs, which shows that after binding with ligands, aldose reductase stability and compactness increase. In the control complex, a consistent RoG value of 19.1 Å was observed, indicating a uniform and stable structure. Similarly, the compounds from the African database showed a comparable RoG pattern to the RMSD results. Specifically, the Diamino-di nitro-methyl dioctyl phthalate-AR complex exhibited a higher RoG pattern, with an average RoG of 19.3 Å. In contrast, the Bisindolylmethane-hydrazone hybrids displayed a lower RoG pattern, consistent with the RMSD results, with an average RoG of 19.0 Å Figures 6A, B. Similarly, the structural compactness of each complex from the in-house database was assessed to examine the variations observed during the simulation. Interestingly, the RoG patterns for the top complexes from the in-house database align strongly with the RMSD results. For instance, the RoG of Bisindolylmethane thiosemicarbazides displayed a uniform RoG value with an average of 10.0 Å. The RoG exhibited a consistent graph with no significant changes in protein size, except for an abrupt decline observed at 40–50 ns. The average RoG for this complex was estimated to be 19.3 Å. In line with the RMSD results for Bisindolylmethane-hydrazone hybrids, the RoG pattern also demonstrated a gradual increase in the RoG Trajectory Figures 6C, D. The top hits from the ZINC database displayed slightly lower but relatively stable RoG values for all the complexes. In the case of the ZINC35671852-AR complex, the RoG initially decreased and reached 19.1 Å at 20 ns It then continued to decrease and stabilised at 19.0 Å for the remaining 100 ns of the simulation. As for the ZINC78774792-AR complex, a consistent and uniform RoG was observed throughout the first 100 ns of the simulation. The average RoG for this complex was calculated to be 19.1 Å Figures 5E, F. In conclusion, the results indicate that the identified hits exhibit stable protein compactness, as reflected by similar average RoG values. Additionally, minimal unbinding events were observed. These findings suggest that these hits have promising pharmacological potential and could potentially serve as effective therapeutics against aldoreductase for the treatment of diabetes.

#### Analysis of hydrogen bond

**FIGURE 7:**
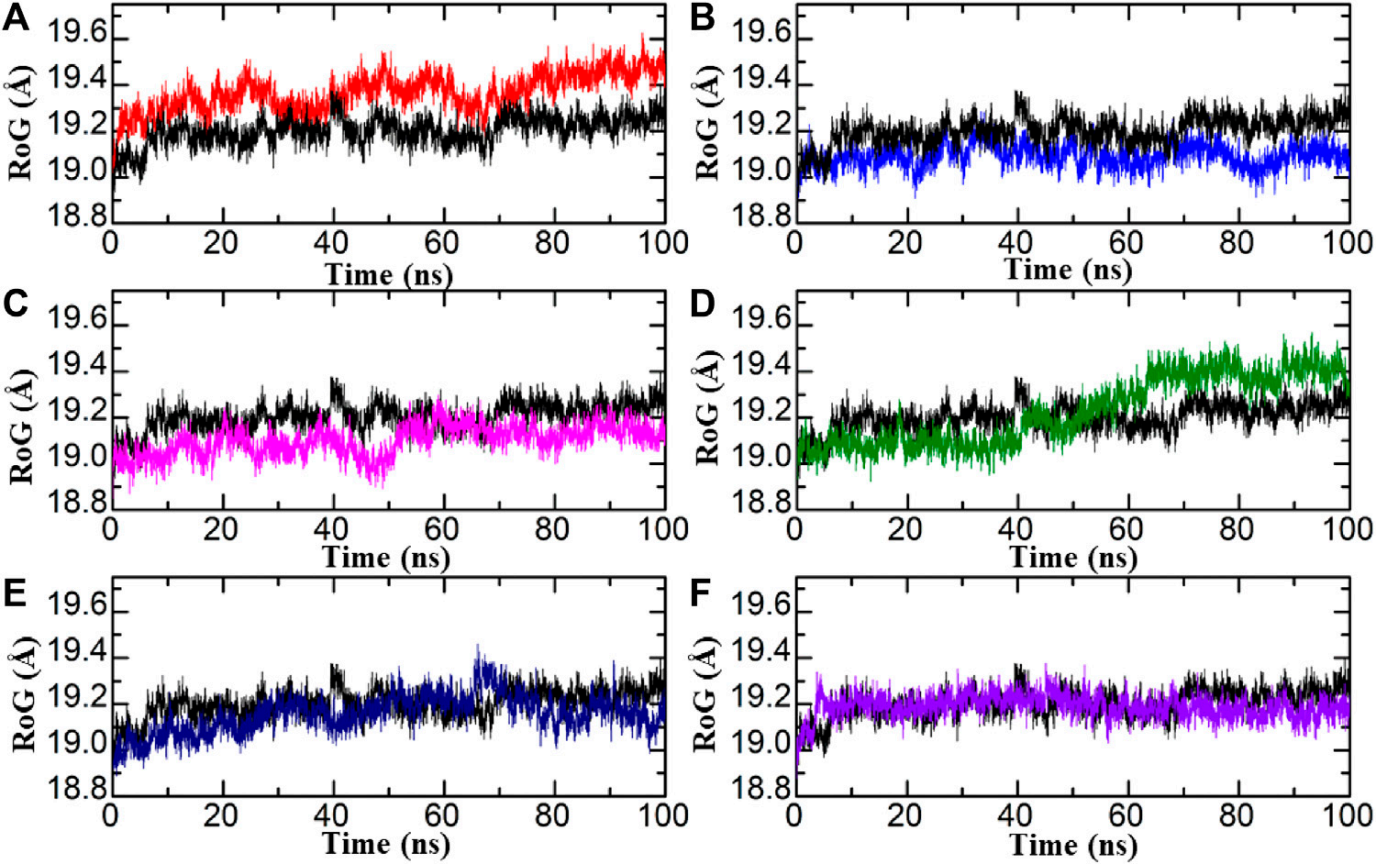
RoG of each hit from four databases in complex with aldoreductase **(A,B)** represent the RMSDs for the tolmetin-aldoreductase and top hits from the African database with aldoreductase. **(C,D)** represent the RoG for the tolmetin-AR and top hits from the in-house database with aldoreductase. **(E,F)** represent the RoG for the tolmetin-aldoreductase and top hits from the ZINC database with aldoreductase.

Hydrogen bond analysis plays an essential role to understand any protein-ligand complex stability. Herein, we studied strength of hydrogen bonds during MD simulation. In current study, we carried out analysis of hydrogen bonds of top two hits from each database for entire 100 ns MD simulation period. As illustrated in Figure 7, the number of hydrogen bonds were increase in all inhibitors from Zinc, South African and in-house databases by comparing with standard drug. The SA1 exist four hydrogen bond where as SA2 mediate three, IH1, IH2, ZN2, and standard drug have number of two hydrogen bonds during MD simulation.

#### Binding free energy calculation

The MMPBSA is one of the commonly employed approaches that is used to access the ligands binding energy with protein molecules. This calculation is an imperative assessment that properly re-evaluates the accuracy and binding conformation of the interacting partner. This approach is the least expensive and has higher accuracy as compared with other techniques. By considering the precious applicability of this approach, we computed the binding free energy for the top inhibitors from each database by utilising MD trajectories. The details of binding free energy are illustrated in Table 2.

**TABLE 2:** Illustration of binding free energy of control drug and finally selected hits.

Complexes	VDW	EEL	ESURF	EGB	ΔG Total	Std. Err. of Mean
*Diamino-di nitro-methyl dioctyl phthalate*	−67.8424	−2.9115	−6.8578	16.9381	−60.6736	0.1813
*Penta-o-galloyl-glucose*	−56.2216	1.2134	−6.2394	10.5192	−50.7283	0.1592
*Bisindolylmethane thiosemi-carbazides*	−52.3106	−0.5548	−6.5891	12.3153	−47.1392	0.1433
Bisindolylmethane–hydrazone hybrids	−61.1410	−4.3809	−7.0350	31.2747	−41.2822	0.2546
*ZINC35671852*	−43.8532	−0.2644	−5.0052	8.9494	−40.1734	0.1046
*ZINC78774792*	−42.0320	−0.8339	−4.8443	8.8884	−38.8218	0.1819
*Control drug* (*Tolmetin*)	−32.0807	−52.8459	−3.5380	64.1539	−24.3108	0.1200

#### Dissociation constant and bioactivity analysis

PRODIGY-LIG generates the KD results as ΔG upon submission of the complex. For the top six hits, the KD values were calculated to be Diamino-di nitro-methyl dioctyl phthalate (−12.44), penta-O-galloyl-glucose (−11.63), Bisindolylmethane thiosemi carbazides (−9.87), Bisindolylmethane–hydrazone hybrids (−10.25), ZINC35671852 (−10.14), and ZINC78774792 (−10.87) respectively. The dissociation constant for the control complex was −8.21, indicating its strong activity against aldoreductase. In terms of bioactivity, a score between −0.5 and 0.5 is considered. A molecule with a bioactivity score greater than 0.00 is likely to possess significant biological activity, while scores between 0.50 and 0.00 indicate moderate activity. Scores below 0.50 are indicative of inactivity. The bioactivity for each of the top hit molecules was estimated to be Diamino-dinitro-methyl dioctyl phthalate (0.15), penta-o-galloyl-glucose (0.27), Bisindolylmethane thiosemi carbazides (0.24), Bisindolylmethane–hydrazone hybrids (0.41), ZINC35671852 (0.23), and ZINC78774792, respectively. The control complex (aldoreductase-tolmetin) was predicted to have a bioactivity score of 0.42, indicating significant biological activity. This suggests that the compounds have a strong potential to inhibit aldoreductase under *in vitro* conditions.

## Conclusion

Different attempts have been made to identify potent drugs against aldose reductase. But none of the drugs show strong efficiency. Therefore, the present study was designed to apply structure-based virtual screening, MD simulation, and the MMPBSA approach to identify a potent drug against aldose reductase. By performing virtual screening, we identified the top six inhibitors from each database, including the South African natural database, the in-house database, and the zinc database, respectively. All these inhibitors were validated and compared with standard drugs through various computational approaches. In these obtained inhibitors, compounds from South Africa and IH1 were found to be the best inhibitors, which presented less carbon alpha deviation, less fluctuation, binding interaction, and a good binding score. However, *in vivo* and *in vitro* analysis were required, which will help validate the activity of compounds for clinical usage.

## Data Availability

The raw data supporting the conclusion of this article will be made available by the authors, without undue reservation.
